# Rapid genomic DNA variation in newly hybridized carp lineages derived from *Cyprinus carpio* (♀) × *Megalobrama amblycephala* (♂)

**DOI:** 10.1186/s12863-019-0784-2

**Published:** 2019-11-28

**Authors:** Kaikun Luo, Shi Wang, Yeqing Fu, Pei Zhou, Xuexue Huang, Qianhong Gu, Wuhui Li, Yude Wang, Fangzhou Hu, Shaojun Liu

**Affiliations:** 1 State Key Laboratory of Developmental Biology of Freshwater Fish, Hunan Normal University, Changsha, 410081 Hunan People’s Republic of China; 2 College of Life Sciences, Hunan Normal University, Changsha, 410081 Hunan People’s Republic of China; 3 College of Chemistry and Chemical Engineering, Hunan Normal University, Changsha, 410081 Hunan People’s Republic of China; 4 Key Laboratory of Tropical and Subtropical Fisheries Resource Application and Cultivation, Ministry of Agriculture, Pearl River Fisheries Research Institute, Chinese Academy of Fishery Sciences, Guangzhou, 510380 Guangdong People’s Republic of China

**Keywords:** Distant hybridization, *Hox* gene, Lineage, Recombinant cluster, Pseudogene

## Abstract

**Background:**

Distant hybridization can generate changes in phenotypes and genotypes that lead to the formation of new hybrid lineages with genetic variation. In this study, the establishment of two bisexual fertile carp lineages, including the improved diploid common carp (IDC) lineage and the improved diploid scattered mirror carp (IDMC) lineage, from the interspecific hybridization of common carp (*Cyprinus carpio*, 2n = 100) (♀) × blunt snout bream (*Megalobrama amblycephala*, 2n = 48) (♂), provided a good platform to investigate the genetic relationship between the parents and their hybrid progenies.

**Result:**

In this study, we investigated the genetic variation of 12 *Hox* genes in the two types of improved carp lineages derived from common carp (♀) × blunt snout bream (♂). *Hox* gene clusters were abundant in the first generation of IDC, but most were not stably inherited in the second generation. In contrast, we did not find obvious mutations in *Hox* genes in the first generation of IDMC, and almost all the *Hox* gene clusters were stably inherited from the first generation to the second generation of IDMC. Interestingly, we found obvious recombinant clusters of *Hox* genes in both improved carp lineages, and partially recombinant clusters of *Hox* genes were stably inherited from the first generation to the second generation in both types of improved carp lineages. On the other hand, some *Hox* genes were gradually becoming pseudogenes, and some genes were completely pseudogenised in IDC or IDMC.

**Conclusions:**

Our results provided important evidence that distant hybridization produces rapid genomic DNA changes that may or may not be stably inherited, providing novel insights into the function of hybridization in the establishment of improved lineages used as new fish resources for aquaculture.

## Background

Hybridization may cause interactions involving a wide range of types and levels of genetic divergence between the parental forms [1]. In nature, hybridization among species is reasonably common on a per-species basis, even though it is usually very rare on a per-individual basis. On a per-individual basis, the isolation mechanisms (e.g., reproductive barriers) prevented the occurrence of high frequency hybridization events among individuals of different species. Although hybrids are rare in populations, a few hybrids can provide a bridge to allow a trickle of alleles to pass between species. Thus, if species that hybridize are common, even low rates of hybridization per individual can have important evolutionary consequences in a high fraction of species. It was found that approximately 10–30% of multicellular animal and plant species hybridize regularly [2]. Hybridization among species can act as an additional, perhaps more abundant, source of adaptive genetic variation than mutation (very rare, approximately 10^− 8^ to 10^− 9^ per generation per base pair) [3–7]. For example, in Darwin’s finches, ‘New additive genetic variance introduced by hybridization is estimated to be two to three orders of magnitude greater than that introduced by mutation’ [3]. In both plants and animals, distant hybridization appears to facilitate speciation and adaptive radiation [8]. Hybridization has played a key role in recombining the adaptive traits of two species and generating novel phenotypes [9]. For example, common wheat (*Triticum aestivum*), originated from hybridization between *T. turgidum* and *Aegilops tauschii*, has significantly increased grain yield and the harvest index [10]; another plant hybrid is derived from the interspecific hybridization between *Vigna umbellata* (♀) and *V. exilis* (♂), which is tolerant to drought and presents early flowering [11]. In Cyprinidae, the autotetraploid hybrids, originated from hybridization between red crucian carp (*Carassius auratus* red *var.*, ♀) × blunt snout bream (*Megalobrama amblycephala*, ♂), has significantly shortened the age of sexual maturity compared to their allotetraploid parents [12]; the hybrids derived from blunt snout bream (♀) × Bleeker’s yellow tail (*Xenocypris davidi* Bleeker, ♂) has showed significantly higher growth rate compared to their parents [13]. Hybridization can lead to rapid genomic changes, including chromosomal rearrangements, genome expansion, genomic DNA variation, differential gene expression, and gene silencing [14]. One such example is that of *Brassica* hybrids, in which multiple genome rearrangements and segment deletions occurred within five generations [15]. In addition, Rieseberg et al. found extensive genomic reorganization and karyotypic evolution in *Helianthus* hybrids, indicating the occurrence of rapid karyotypic evolution [16]. In Cyprinidae, in the allotetraploid hybrids, chimeric genes (9.67–11.06%) and mutation events (1.02–1.16%) occurred in different generations of this nascent allopolyploids [17]; Liu et al. revealed 19.04%, 4.17% chimeric genes and 6.90%, 5.05% mutations of orthologous genes in F_1_ and F_2_ of diploid hybrids, respectively [18]. Distant hybridization can generate changes in phenotypes and genotypes, leading to the formation of new hybrid lineages with genetic variation and providing a good experimental model for tracing the changes of genetic and epigenetic levels in the early stage of distant hybridization. Moreover, these newly established bisexual fertile diploid and tetraploid lineages provide new germplasm resources, which are used to produce improved diploid and triploid varieties by crossing diploid species, respectively [19–22].

**Fig. 1:**
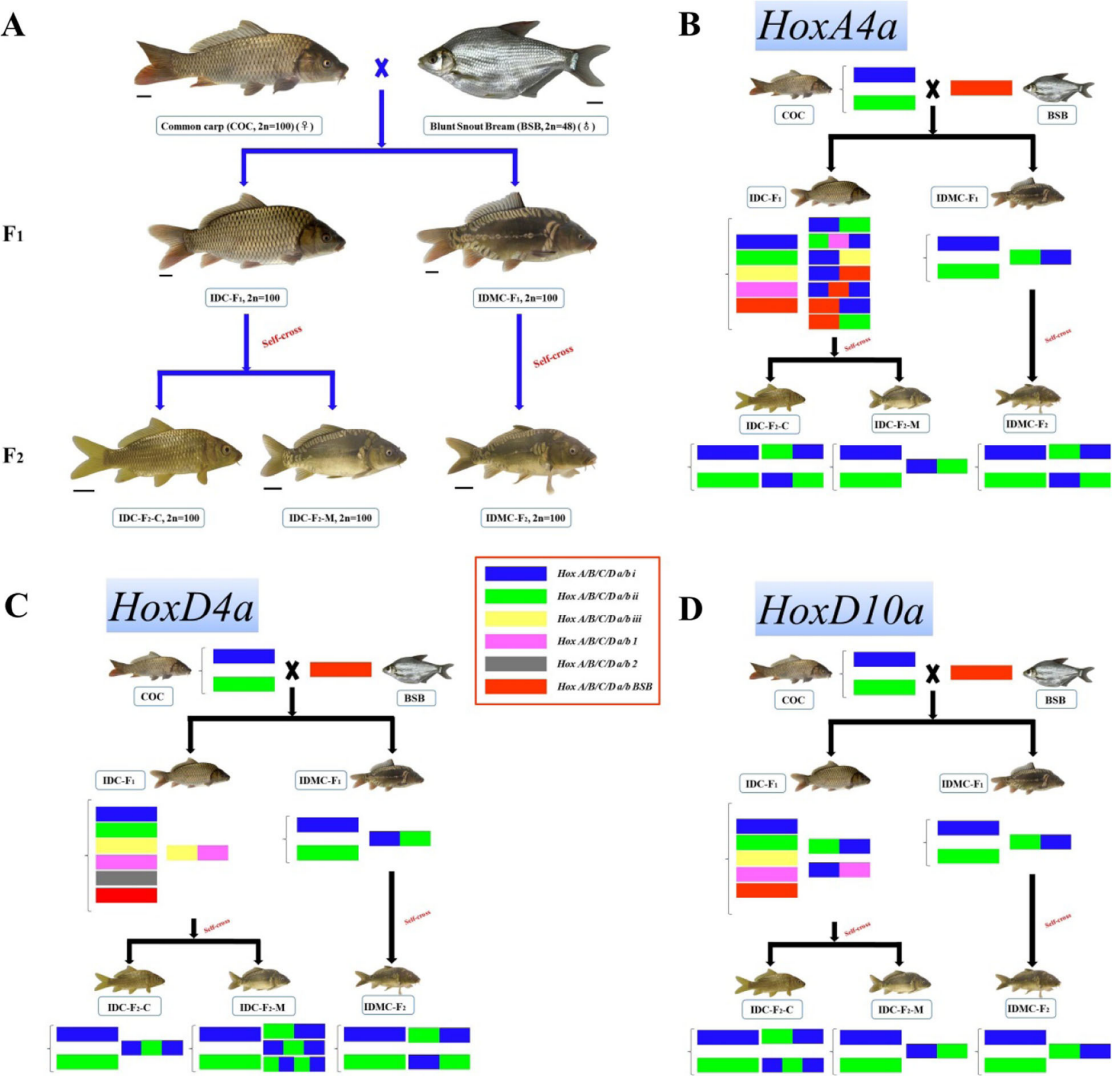
Crossing procedure and appearances of COC, BSB, IDC-F_1_, IDMC-F_1_, IDC-F_2_-C, IDC-F_2_-M, and IDMC-F_2_; variable sequence types (including haplotypes and recombinant clusters) in different *Hox* genes in these species. **a** Crossing procedure and appearances of COC, BSB, IDC-F_1_, IDMC-F_1_, IDC-F_2_-C, IDC-F_2_-M, and IDMC-F_2_. **b** Variable sequence types (including haplotypes and recombinant clusters) in *HoxA4a* in these species. **c** Variable sequence types (including haplotypes and recombinant clusters) in *HoxD4a* in these species. **d** Variable sequence types (including haplotypes and recombinant clusters) in *HoxD10a* in these species

In our previous study, we successfully obtained two types of improved carp offspring from common carp (2n = 100, abbreviated COC) (♀) × blunt snout bream (2n = 48, abbreviated BSB) (♂); one is the improved diploid common carp (2n = 100, IDC-F_1_), and the other is the improved diploid scattered mirror carp (2n = 100, IDMC-F_1_) [23]. In this study, we carried out self-crossing of these two types of improved carp offspring (IDC-F_1_ and IDMC-F_1_), respectively. Interestingly, the self-crossed offspring of IDC-F_1_ showed two phenotypes: one was consistent with that of their parents (abbreviated IDC-F_2_-C), and the other was very similar to that of IDMC-F_1_ (abbreviated IDC-F_2_-M) (Fig. 1a). In contrast, the self-crossed offspring of IDMC-F_1_ showed only one phenotype, that is, a scattered mirror carp-like appearance (abbreviated IDMC-F_2_) (Fig. 1a). To further explore the relationship of the genetic evolution of COC, IDC and IDMC, we studied the *Hox* gene structures in the genomic DNA of the different generations of the IDC and IDMC lineages. Determination of the genotypes of these lineages is very useful for understanding the processes associated with the genomic DNA changes that accompany phenotype changes.

*Hox* genes, which encode transcription factors, are essential for the development of various morphological features. In vertebrates, *Hox* genes consist of two exons and the highly conserved homeodomain (60 aa), which is encoded by the second exon [24]. Late evolutionary novelties are generally considered to be associated either with the emergence of particular lineages or with important steps in their unique evolution [25]. Recent studies have shown that the origin and evolution of the *Hox* genes played a crucial role in genome replication, sequence variation, and selective pressure [25–28]. The search for regulatory elements through comparative genomic approaches using *Hox* genes promises to be particularly successful because their nucleotide sequences and functions are extremely conserved in all vertebrates; meanwhile, *Hox* gene clusters provide a good starting point for the study of genetic variation in genomic DNA [29].

## Results

### Sequence information for COC, BSB, IDC-F_1_, IDMC-F_1_, IDC-F_2_-C, IDC-F_2_-M, and IDMC-F_2_ clones

In this study, we used 12 pairs of degenerate PCR primers (Additional file 1: Table S1) to obtain partial sequence information for 20 putative *Hox* genes from COC, 12 putative *Hox* genes from BSB, 42 putative and 15 recombinant *Hox* genes from IDC-F_1_, 19 putative and 5 recombinant *Hox* genes from IDMC-F_1_, 17 putative and 12 recombinant *Hox* genes from IDMC-F_2_, 19 putative and 10 recombinant *Hox* genes from IDC-F_2_-C, and 18 putative and 13 recombinant *Hox* genes from IDC-F_2_-M. All of these fragments were between 700 and 1600 bp in length, including the exon 1-intron-exon 2 region (Tables 1 and 2). In this study, to avoid biased amplification of only one copy of the characterized *Hox* genes, we selected 30 clones of each gene from IDC-F_1_, IDMC-F_1_, IDC-F_2_-C, IDC-F_2_-M, and IDMC-F_2_ and 20 clones of each gene from COC and BSB. All fragments from COC, BSB, IDC-F_1_, IDMC-F_1_, IDC-F_2_-C, IDC-F_2_-M, and IDMC-F_2_ were confirmed to be *Hox* gene sequences via the NCBI website (http://www.ncbi.nlm.nih.gov), and each included the conserved homeobox region. All of the sequence information and GenBank accession numbers in this study is detailed in Additional file 1: Table S2.

**Table 1:** PCR amplification bands (non-recombinant bands) in COC, BSB, IDC-F_1_, IDMC-F_1_, IDMC-F_2_, IDC-F_2_-C, and IDC-F_2_-M

Genes	Species	Locus	Size (bp)	Exon 1 (bp)	Intron (bp)	Exon 2 (bp)
HoxA4a	COC	*HoxA4ai*	1177	89–500	501–970	971–1177
		*HoxA4aii*	1182	89–500	501–975	976–1182
	BSB	*HoxA4a-BSB*	1188	89–500	501–981	982–1188
	IDC-F_1_	*HoxA4ai*	1177	89–500	501–970	971–1177
		*HoxA4aii*	1182	89–500	501–975	976–1182
		*HoxA4aiii*	1184	89–500	501–977	978–1184
		*HoxA4a-1*	1181	89–500	501–974	975–1181
		*HoxA4a-BSB*	1188	89–500	501–981	982–1188
	IDMC-F_1_	*HoxA4ai*	1177	89–500	501–970	971–1177
		*HoxA4aii*	1182	89–500	501–975	976–1182
	IDMC-F_2_	*HoxA4ai*	1177	89–500	501–970	971–1177
		*HoxA4aii*	1182	89–500	501–975	976–1182
	IDC-F_2_-C	*HoxA4ai*	1177	89–500	501–970	971–1177
		*HoxA4aii*	1182	89–500	501–975	976–1182
	IDC-F_2_-M	*HoxA4ai*	1177	89–500	501–970	971–1177
		*HoxA4aii*	1182	89–500	501–975	976–1182
HoxA9a	COC	*HoxA9ai*	817	1–381	382–620	621–817
		*HoxA9aii*	891	1–381	382–694	695–891
	BSB	*HoxA9b*	879	1–381	382–682	683–879
	IDC-F_1_	*HoxA9ai*	817	1–381	382–620	621–817
		*HoxA9aii*	867	1–381	382–670	671–867
	IDMC-F_1_	*HoxA9ai*	817	1–381	382–620	621–817
	IDMC-F_2_	*HoxA9ai*	817	1–381	382–620	621–817
	IDC-F_2_-C	*HoxA9ai*	817	1–381	382–620	621–817
		*HoxA9aii*	891	1–381	382–694	695–891
	IDC-F_2_-M	*HoxA9ai*	817	1–381	382–620	621–817
HoxA2b	COC	*HoxA2bi*	1490	1–314	315–905	906–1490
		*HoxA2bii*	1475	1–314	315–890	891–1475
	BSB	*HoxA2b*	1479	1–311	312–894	895–1479
	IDC-F_1_	*HoxA2bi*	1490	1–314	315–905	906–1490
		*HoxA2bii*	1475	1–314	315–890	891–1475
		*HoxA2biii*	1486	1–314	315–901	902–1486
		*HoxA2b-1*	1448	1–314	315–863	864–1448
	IDMC-F_1_	*HoxA2bi*	1490	1–314	315–905	906–1490
		*HoxA2bii*	1475	1–314	315–890	891–1475
	IDC-F_2_-C	*HoxA2bi*	1490	1–314	315–905	906–1490
		*HoxA2bii*	1475	1–314	315–890	891–1475
	IDC-F_2_-M	*HoxA2bi*	1490	1–314	315–905	906–1490
		*HoxA2bii*	1475	1–314	315–890	891–1475
	IDMC-F_2_	*HoxA2bi*	1490	1–314	315–905	906–1490
		*HoxA2bii*	1475	1–314	315–890	891–1475
HoxA11b	COC	*HoxA11bi*	1440	3–590	591–1342	1343–1440
	BSB	*HoxA11b-BSB*	1703	3–602	603–1605	1606–1703
	IDC-F_1_	*HoxA11bi*	1440	3–590	591–1342	1343–1440
		*HoxA11bii*	1401	3–590	591–1303	1304–1401
	IDMC-F_1_	*HoxA11bi*	1439	3–590	591–1342	1343–1439
	IDMC-F_2_	*HoxA11bi*	1439	3–590	591–1342	1343–1439
	IDC-F_2_-C	*HoxA11bi*	1440	3–590	591–1342	1343–1440
	IDC-F_2_-M	*HoxA11bi*	1440	3–590	591–1342	1343–1440
HoxB1a	COC	*HoxB1ai*^ψ^	1510	–	–	–
		*HoxB1aii*	1526	1–462	463–1250	1251–1526
	BSB	*HoxB1a*	1522	1–459	460–1246	1247–1522
	IDC-F_1_	*HoxB1ai*^ψ^	1510	–	–	–
		*HoxB1aiii*	1484	1–450	451–1208	1209–1484
	IDMC-F_1_	*HoxB1ai*^ψ^	1510	–	–	–
		*HoxB1aii*	1525	1–462	463–1249	1250–1525
	IDMC-F_2_	*HoxB1ai*^ψ^	1510	–	–	–
	IDC-F_2_-C	*HoxB1ai*^ψ^	1510	–	–	–
	IDC-F_2_-M	*HoxB1ai*^ψ^	1510	–	–	–
HoxB4a^ψ^	COC	*HoxB4ai*^ψ^	1631	–	–	–
	BSB	*HoxB4a*^ψ^	1617	–	–	–
	IDC-F_1_	*HoxB4ai*^ψ^	1630	–	–	–
		*HoxB4aii*^ψ^	1613	–	–	–
	IDMC-F_1_	*HoxB4ai*^ψ^	1630	–	–	–
	IDMC-F_2_	*HoxB4ai*^ψ^	1630	–	–	–
	IDC-F_2_-C	*HoxB4ai*^ψ^	1630	–	–	–
	IDC-F_2_-M	*HoxB4ai*^ψ^	1630	–	–	–
HoxB1b	COC	*HoxB1bi*	731	1–477	478–565	566–731
	BSB	*HoxB1b-BSB*	751	1–477	478–585	586–751
	IDC-F_1_	*HoxB1bi*	731	1–477	478–565	566–731
		*HoxB1bii*	733	1–477	478–567	568–733
		*HoxB1b-BSB*	751	1–477	478–585	586–751
	IDMC-F_1_	*HoxB1bi*	731	1–477	478–565	566–731
	IDMC-F_2_	*HoxB1bi*	731	1–477	478–565	566–731
	IDC-F_2_-C	*HoxB1bi*	731	1–477	478–565	566–731
	IDC-F_2_-M	*HoxB1bi*	731	1–477	478–565	566–731
HoxB5b	COC	*HoxB5bi*	1191	1–561	562–985	986–1191
		*HoxB5bii*	1190	1–564	565–984	985–1190
	BSB	*HoxB5b-BSB*	1227	1–564	565–1021	1022–1227
	IDC-F_1_	*HoxB5bi*	1191	1–561	562–985	986–1191
		*HoxB5bii*	1190	1–564	565–984	985–1190
		*HoxB5biii*	1196	1–561	562–990	991–1196
		*HoxB5b-BSB*	1226	1–564	565–1020	1021–1226
	IDMC-F_1_	*HoxB5bi*	1191	1–561	562–985	986–1191
		*HoxB5bii*	1190	1–564	565–984	985–1190
	IDMC-F_2_	*HoxB5bi*	1191	1–561	562–985	986–1191
	IDC-F_2_-C	*HoxB5bi*	1190	1–561	562–984	985–1190
		*HoxB5bii*	1190	1–564	565–984	985–1190
	IDC-F_2_-M	*HoxB5bi*	1190	1–561	562–984	985–1190
		*HoxB5bii*	1190	1–564	565–984	985–1190
HoxC4a	COC	*HoxC4ai*	1169	1–410	411–928	929–1169
		*HoxC4aii*	1176	1–410	411–935	936–1176
	BSB	*HoxC4a*	1125	1–410	411–933	934–1125
	IDC-F_1_	*HoxC4ai*	1169	1–410	411–928	929–1169
		*HoxC4aii*	1176	1–410	411–935	936–1176
		*HoxC4aiii*	1173	1–410	411–932	933–1173
		*HoxC4a-1*	1179	1–410	411–938	939–1179
	IDMC-F_1_	*HoxC4ai*	1169	1–410	411–928	929–1169
		*HoxC4aii*	1176	1–410	411–935	936–1176
	IDMC-F_2_	*HoxC4ai*	1168	1–410	411–928	929–1168
		*HoxC4aii*	1174	1–410	411–934	935–1174
	IDC-F_2_-C	*HoxC4ai*	1169	1–410	411–928	929–1169
		*HoxC4aii*	1176	1–410	411–935	936–1176
	IDC-F_2_-M	*HoxC4ai*	1169	1–410	411–928	929–1169
		*HoxC4aii*	1175	1–410	411–934	935–1175
HoxC6b	COC	*HoxC6bi*	942	2–392	393–763	764–942
	BSB	*HoxC6b-BSB*	922	2–392	393–737	738–922
	IDC-F_1_	*HoxC6bi*	949	2–392	393–763	764–949
		*HoxC6bii*	964	2–392	393–778	779–964
		*HoxC6b-BSB*	923	2–392	393–737	738–923
	IDMC-F_1_	*HoxC6bi*	949	2–392	393–763	764–949
	IDMC-F_2_	*HoxC6bi*	949	2–392	393–763	764–949
	IDC-F_2_-C	*HoxC6bi*	949	2–392	393–763	764–949
	IDC-F_2_-M	*HoxC6bi*	949	2–392	393–763	764–949
HoxD4a	COC	*HoxD4ai*	942	1–315	316–717	718–942
		*HoxD4aii*	944	1–315	316–719	720–944
	BSB	*HoxD4a-BSB*	911	1–306	307–686	687–911
	IDC-F_1_	*HoxD4ai*	942	1–315	316–717	718–942
		*HoxD4aii*	944	1–315	316–719	720–944
		*HoxD4aiii*	952	1–315	316–727	728–952
		*HoxD4a-1*	937	1–315	316–712	713–937
		*HoxD4a-2*	960	1–315	316–735	736–960
		*HoxD4a-BSB*	911	1–306	307–686	687–911
	IDMC-F_1_	*HoxD4ai*	942	1–315	316–717	718–942
		*HoxD4aii*	944	1–315	316–719	720–944
	IDMC-F_2_	*HoxD4ai*	942	1–315	316–717	718–942
		*HoxD4aii*	944	1–315	316–719	720–944
	IDC-F_2_-C	*HoxD4ai*	942	1–315	316–717	718–942
		*HoxD4aii*	944	1–315	316–719	720–944
	IDC-F_2_-M	*HoxD4ai*	942	1–315	316–717	718–942
		*HoxD4aii*	944	1–315	316–719	720–944
HoxD10a	COC	*HoxD10ai*	1551	1–589	590–1321	1322–1551
		*HoxD10aii*	1546	1–592	593–1316	1317–1546
	BSB	*HoxD10a-BSB*	1574	1–592	593–1344	1345–1574
	IDC-F_1_	*HoxD10ai*	1554	1–589	590–1324	1325–1554
		*HoxD10aii*	1546	1–592	593–1316	1317–1546
		*HoxD10aiii*	1495	1–592	593–1265	1266–1495
		*HoxD10a-1*	1480	1–592	593–1250	1251–1480
		*HoxD10a-BSB*	1574	1–592	593–1344	1345–1574
	IDMC-F_1_	*HoxD10ai*	1554	1–589	590–1324	1325–1554
		*HoxD10aii*	1546	1–592	593–1316	1317–1546
	IDMC-F_2_	*HoxD10ai*	1554	1–589	590–1324	1325–1554
		*HoxD10aii*	1546	1–592	593–1316	1317–1546
	IDC-F_2_-C	*HoxD10ai*	1554	1–589	590–1324	1325–1554
		*HoxD10aii*	1546	1–592	593–1316	1317–1546
	IDC-F_2_-M	*HoxD10ai*	1554	1–589	590–1324	1325–1554
		*HoxD10aii*	1544	1–592	593–1314	1315–1544

^Ψ^denotes a pseudogene

**Table 2:** PCR amplification bands (recombinant bands) in COC, BSB, IDC-F_1_, IDMC-F_1_, IDMC-F_2_, IDC-F_2_-C, and IDC-F_2_-M

Genes	Species	Locus	Size (bp)	Exon1 (bp)	Intron (bp)	Exon2 (bp)
HoxA4a	IDC-F1	*HoxA4ai + HoxA4aii*	1182	89–500	501–975	976–1182
		*HoxA4aii + HoxA4a-1 + HoxA4ai*	1182	89–500	501–975	976–1182
		*HoxA4ai + HoxA4aiii*	1184	89–500	501–977	978–1184
		*HoxA4ai + HoxA4a-BSB*	1188	89–500	501–981	982–1188
		*HoxA4ai + HoxA4a-BSB + HoxA4ai*	1182	89–500	501–975	976–1182
		*HoxA4a-BSB + HoxA4ai*	1182/1188	89–500	501–975/981	976/982–1182/1188
		*HoxA4a-BSB + HoxA4aii*	1188	89–500	501–981	982–1188
	IDMC-F1	*HoxA4aii + HoxA4ai*	1182	89–500	501–975	976–1182
	IDMC-F2	*HoxA4aii + HoxA4ai*	1182	89–500	501–975	976–1182
		*HoxA4ai + HoxA4aii*	1182	89–500	501–975	976–1182
	IDC-F2-C	*HoxA4aii + HoxA4ai*	1177/1182	89–500	501–970/975	971/976–1177/1182
		*HoxA4ai + HoxA4aii*	1177/1182	89–500	501–970/975	971/976–1177/1182
	IDC-F2-M	*HoxA4ai + HoxA4aii*	1177/1182	89–500	501–970/975	971/976–1177/1182
HoxA9a	IDMC-F2	*HoxA9ai + HoxA9aii*	891	1–381	382–694	695–891
HoxA2b	IDC-F1	*HoxA2bi + HoxA2bii*	1490	1–314	315–905	906–1490
	IDMC-F2	*HoxA2bi + HoxA2bii*	1490	1–314	315–905	906–1490
	IDC-F2-C	*HoxA2bii + HoxA2bi*	1475	1–314	315–890	891–1475
	IDC-F2-M	*HoxA2bii + HoxA2bi*	1475	1–314	315–890	891–1475
HoxA11b	IDMC-F2	*HoxA11bi + HoxA11b-BSB + HoxA11bi*	1439/1455	3–590/605	591/606–1342/1358	1343/1359–1439/1455
	IDC-F2-M	*HoxA11bi + HoxA11b-BSB + HoxA11bi*	1455	3–605	606–1357	1358–1455
HoxB1a	IDC-F2-M	*HoxB1ai + HoxB1aii*^ψ^	1510	1–462	463–1234	1235–1510
		*HoxB1aii + HoxB1ai*^ψ^	1503	1–462	463–1227	1228–1503
HoxB5b	IDC-F1	*HoxB5bi + HoxB5bii + HoxB5bi*	1190	1–564	565–984	985–1190
		*HoxB5bi + HoxB5bii*	1190	1–564	565–984	985–1190
	IDMC-F1	*HoxB5bii + HoxB5bi*	1194	1–564	565–988	989–1194
	IDMC-F2	*HoxB5bi + HoxB5bii*	1187	1–561	562–981	982–1187
		*HoxB5bii + HoxB5bi + HoxB5bii*	1194	1–564	565–988	989–1194
	IDC-F2-C	*HoxB5bii + HoxB5bi*	1190	1–561	562–984	985–1190
	IDC-F2-M	*HoxB5bii + HoxB5bi*	1190	1–564	565–984	985–1190
		*HoxB5bi + HoxB5bii*	1188	1–561	562–982	983–1188
HoxC4a	IDC-F1	*HoxC4aii + HoxC4ai*	1174	1–410	411–933	934–1174
		*HoxC4aii + HoxC4aiii*	1173	1–410	411–932	933–1173
	IDMC-F1	*HoxC4aii + HoxC4ai*^ψ^	1169	1–410	411–928	929–1169
	IDMC-F2	*HoxC4ai + HoxC4aii*	1168/1175	1–410	411–928/935	929/936–1168/1175
		*HoxC4aii + HoxC4ai*	1168	1–410	411–928	929–1168
	IDC-F2-C	*HoxC4ai + HoxC4aii*	1169/1175	1–410	411–928/934	929/935–1169/1175
		*HoxC4aii + HoxC4ai*	1169	1–410	411–928	929–1169
		*HoxC4ai + HoxC4aii + HoxC4ai*	1175	1–410	411–934	935–1175
	IDC-F2-M	*HoxC4ai + HoxC4aii*	1175	1–410	411–934	935–1175
		*HoxC4aii + HoxC4ai*	1175	1–410	411–934	935–1175
HoxD4a	IDC-F1	*HoxD4aiii + HoxD4a-1*	937	1–315	316–712	713–937
	IDMC-F1	*HoxD4ai + HoxD4aii*	944	1–315	316–719	720–944
	IDMC-F2	*HoxD4ai + HoxD4aii*	944	1–315	316–719	720–944
		*HoxD4aii + HoxD4ai*	942	1–315	316–717	718–942
	IDC-F2-C	*HoxD4ai + HoxD4aii + HoxD4ai*	936	1–314	315–711	712–936
	IDC-F2-M	*HoxD4aii + HoxD4ai*	937/944	1–315	316–712/719	713/720–937/944
		*HoxD4ai + HoxD4aii + HoxD4ai*	937	1–315	316–712	713–937
		*HoxD4aii + HoxD4ai + HoxD4aii + HoxD4ai*	937	1–315	316–712	713–937
HoxD10a	IDC-F1	*HoxD10aii + HoxD10ai*	1554	1–589	590–1324	1325–1554
		*HoxD10ai + HoxD10a-1*	1494	1–588	589–1264	1265–1494
	IDMC-F1	*HoxD10aii + HoxD10ai*	1545	1–592	593–1315	1316–1545
	IDMC-F2	*HoxD10aii + HoxD10ai*	1542/1558	1–592	593–1312/1329	1313/1330–1542/1558
	IDC-F2-C	*HoxD10aii + HoxD10ai*	1556	1–592	593–1326	1327–1556
		*HoxD10ai + HoxD10aii + HoxD10ai*	1545	1–592	593–1315	1316–1545
	IDC-F2-M	*HoxD10ai + HoxD10aii*	1544/1554	1–589	590–1314/1324	1315/1325–1544/1554

^Ψ^denotes a pseudogene

### Molecular organization of the *Hox* genes sequences

**Fig. 2:**
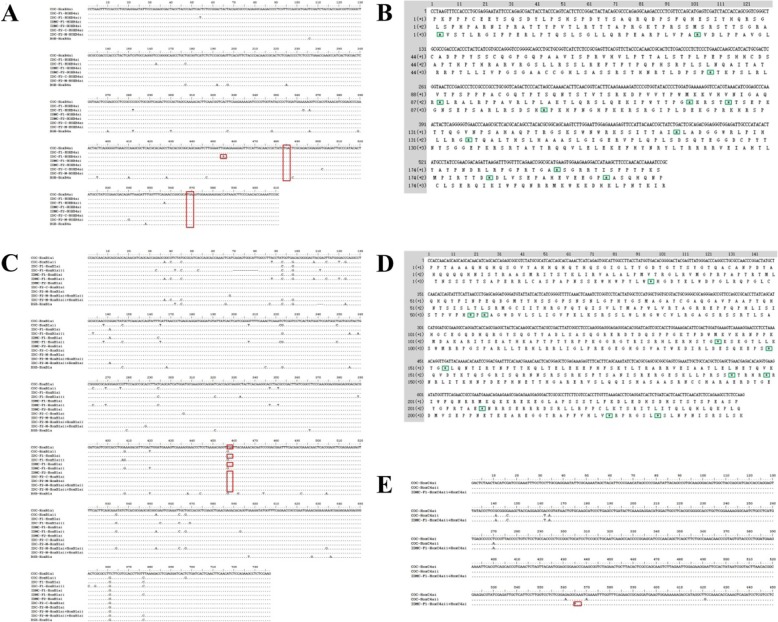
Pseudogene sequences of *HoxB1a*, *HoxB4a*, and *HoxC4a*. **a** The nucleotide sequences of *HoxB4a* in COC, BSB, IDC-F_1_, IDMC-F_1_, IDC-F_2_-C, IDC-F_2_-M, and IDMC-F_2_. **b** The putative amino acid sequence of *HoxB4ai* in COC. **c** The nucleotide sequences of *HoxB1a* in COC, BSB, IDC-F_1_, IDMC-F_1_, IDC-F_2_-C, IDC-F_2_-M, and IDMC-F_2_. **d** The putative amino acid sequence of *HoxB1ai* in COC. **e** The nucleotide sequences of *HoxC4a* in COC and IDMC-F_1_. The red boxes indicate the stop codon bases. The green boxes indicate that there is no corresponding amino acid site due to the occurrence of the stop codon, and the “*” sign is used instead

The organization of the *Hox* clusters in COC, BSB, IDC-F_1_, IDMC-F_1_, IDC-F_2_-C, IDC-F_2_-M, and IDMC-F_2_ are shown in Tables 1 and 2. Figure 1 and Additional file 2: Figures S1-S3 visually reflect the genetic variation in the *Hox* gene clusters of the two types of improved carp lineages. The *Hox* gene cluster organization showed that, as the first generation of distant hybridization, IDC-F_1_ had undergone extremely significant mutations; for example, in *HoxA4a*, IDC-F_1_ has five putative clusters and seven recombinant clusters (Fig. 1b and Tables 1 and 2); in *HoxD4a*, IDC-F_1_ has six putative clusters and one recombinant cluster (Fig. 1c and Tables 1 and 2); in *HoxD10a*, IDC-F_1_ has five putative clusters and two recombinant clusters (Fig. 1d and Tables 1 and 2). However, most of the *Hox* gene clusters in IDC-F_1_ (with a total of 42 putative and 15 recombinant *Hox* gene clusters) were not stably inherited in the second generation (IDC-F_2_-C and IDC-F_2_-M): IDC-F_2_-C has only 19 putative and 10 recombinant *Hox* gene clusters (Fig. 1, Additional file 2: Figures S1-S3 and Tables 1 and 2), and IDC-F_2_-M has only 18 putative and 13 recombinant *Hox* gene clusters (Fig. 1, Additional file 2: Figures S1-S3 and Tables 1 and 2). Additionally, although it was the first generation of a distant hybridization, we did not find obvious mutations in the *Hox* genes of the first generation of IDMC; almost all of the *Hox* gene clusters were derived from the female parent, COC, except for five recombinant clusters (Fig. 1, Additional file 2: Figures S1-S3 and Tables 1 and 2). Almost all of the *Hox* gene clusters in IDMC-F_1_ were stably inherited by the second generation (IDMC-F_2_), but IDMC-F_2_ had more obvious recombination events (the number of recombinant clusters increased to 12) (Fig. 1, Additional file 2: Figures S1-S3 and Tables 1 and 2). In this study, the self-crossed offspring of IDC-F_1_ showed two phenotypes. One of the offspring, IDC-F_2_-M, had a similar phenotype to that of IDMC-F_1_, so we searched for similarities and differences among the *Hox* gene clusters between the offspring IDC-F_2_-M and IDMC-F_1_ or IDMC-F_2_. Notably, we found similarities in the *Hox* gene clusters of these species (Fig. 1, Additional file 2: Figures S1-S3 and Tables 1 and 2); for example, the type of recombinant cluster including *HoxA11b* (*HoxA11bi* + *HoxA11b-BSB* + *HoxA11bi*) was found in only IDC-F_2_-M and IDMC-F_2_ (Additional file 2: Figure S1 c). In addition, as shown in Fig. 1, Additional file 2: Figures S1-S3 and Tables 1 and 2, IDC-F_2_-M possessed more abundant *Hox* gene clusters than IDMC-F_1_, similar to IDMC-F_2_, except that the *Hox* genes of IDMC-F_2_ were mainly concentrated in recombinant clusters. Among these *Hox* gene clusters, we found that all copies of *HoxB4a* in COC, BSB, IDC-F_1_, IDMC-F_1_, IDC-F_2_-C, IDC-F_2_-M, and IDMC-F_2_ were pseudogenes containing a stop codon that prematurely terminates the expression of a full-length functional product (Fig. 2a, b and Tables 1 and 2). We also found that the copies of *HoxB1ai* in COC, IDC-F_1_, IDMC-F_1_, IDC-F_2_-C, IDC-F_2_-M, and IDMC-F_2_ were pseudogenes due to stop codons (Fig. 2c, d and Tables 1 and 2). These results revealed that the *Hox* gene family in cyprinid fishes had undergone rapid evolution, with some genes gradually becoming pseudogenes, and some genes completely pseudogenised. Moreover, we also found pseudogenes in the recombinant clusters; for example, *HoxB1ai + HoxB1aii* and *HoxB1aii* + *HoxB1ai* in IDC-F_2_-M (Fig. 2c and Tables 1 and 2) and *HoxC4aii + HoxC4ai* in IDMC-F_1_ (Fig. 2e and Tables 1 and 2).

### Phylogenetic relationships

**Fig. 3:**
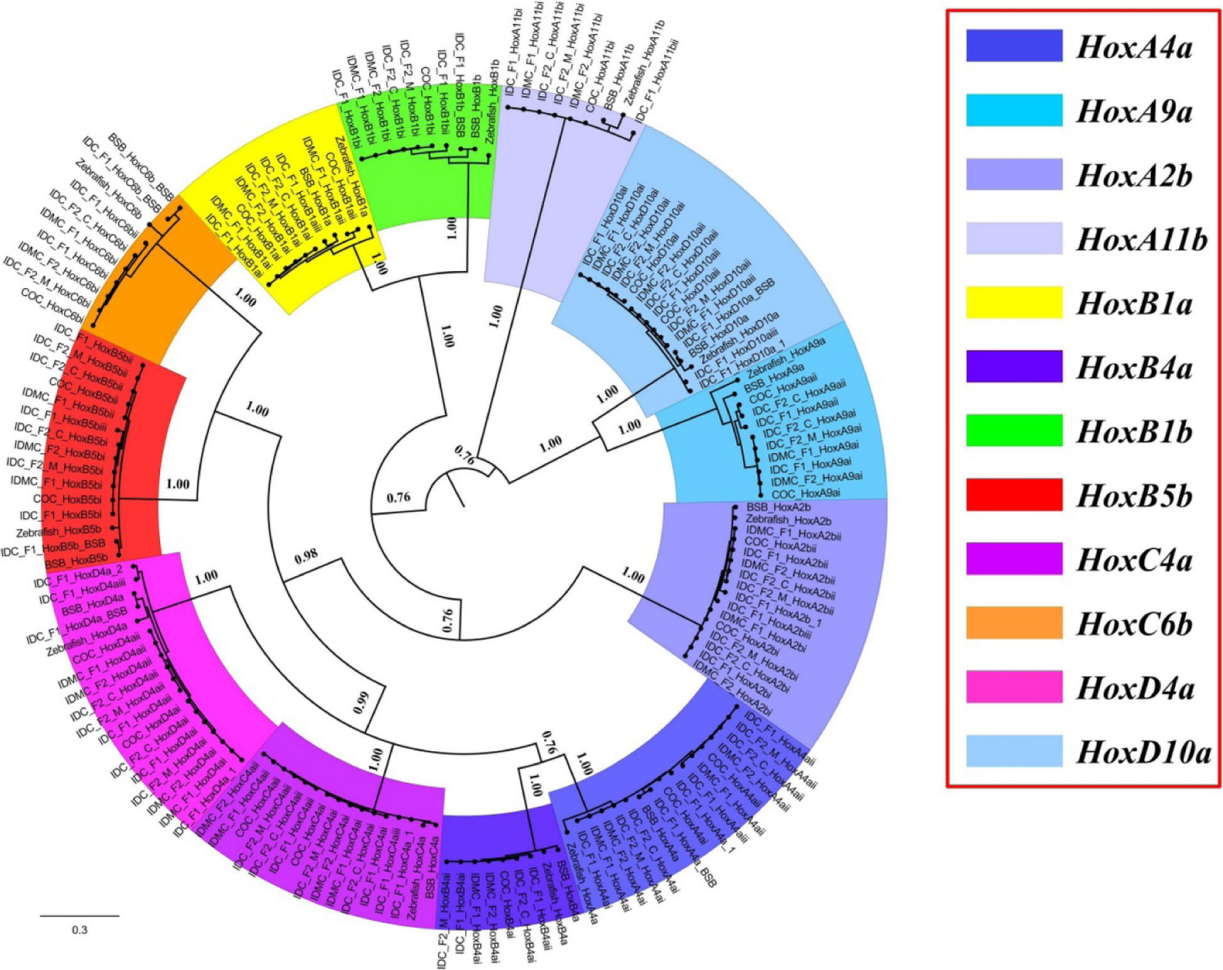
Phylogenetic analyses of the amino acid sequences of 12 *Hox* genes (*HoxA4a*, *HoxA9a*, *HoxA2b*, *HoxA11b*, *HoxB1a*, *HoxB4a*, *HoxB1b*, *HoxB5b*, *HoxC4a*, *HoxC6b*, *HoxD4a*, and *HoxD10a*) in COC, BSB, IDC-F_1_, IDMC-F_1_, IDC-F_2_-C, IDC-F_2_-M, IDMC-F_2_ and zebrafish (*Danio rerio*). Phylogenetic tree constructed using MrBayes with the HKY + I + G model (−lnL = 15,356.5967); MCMC = 2 million generations. The phylogenetic tree for each *Hox* gene is marked by a separate colour, as shown in the figure

An unrooted phylogenetic tree of 12 *Hox* genes was constructed using MrBayes based on the alignment results (Fig. 3). The overall phylogenetic tree was divided into twelve well-conserved clades, and each clade contained one zebrafish *Hox* gene. Meanwhile, we analysed the percentage nucleotide identity and the percentage amino acid identity between duplicated *Hox* coding regions in COC, BSB, IDC-F_1_, IDMC-F_1_, IDMC-F_2_, IDC-F_2_-C, and IDC-F_2_-M (Tables 3 and 4). As shown in Tables 3 and 4, the close relationships were observed among IDC-F_1_, IDC-F_2_-C, and IDC-F_2_-M within the IDC lineage; between IDMC-F_1_ and IDMC-F_2_ within the IDMC lineage; and among IDC-F_1_, IDMC-F_1_, IDMC-F_2_, IDC-F_2_-C, and IDC-F_2_-M within both lineages. To evaluate the speciation of the two types of improved carp lineages, the percentages of nucleotide (amino acid) identity among the 12 *Hox* gene groups in COC, BSB, and both improved carp lineages were examined (Tables 3 and 4, Fig. 3). The identities of the orthologous *Hox* genes between the two types of improved carp lineages and COC were much higher than those between the two types of improved carp lineages and BSB, except for the gene clusters inherited from BSB. In some *Hox* genes, such as *HoxA4a*, *HoxA2b* and *HoxC4a*, both the nucleotide and amino acid sequences of both improved carp lineages had a high degree of identity to COC and BSB. In some *Hox* genes, such as *HoxA11b*, *HoxC6b*, *HoxD4a* and *HoxD10a*, although the nucleotide sequences between the two types of improved carp lineages and COC or BSB had lower identities, they had higher amino acid sequence identities, which suggested that most mutations were synonymous. In some *Hox* genes, such as *HoxA9a*, both the nucleotide and amino acid sequences of both improved carp lineages had a low degree of identity to COC and BSB (Tables 3 and 4).

**Table 3:** Percentage nucleotide identity (on the left) and percentage amino acid identity (on the right) between duplicated *Hox* coding regions in COC, BSB, IDC-F_1_, IDMC-F_1_, IDMC-F_2_, IDC-F_2_-C, and IDC-F_2_-M (following the format below)

	*HoxA4a* (%)	*HoxA9a* (%)	*HoxA2b* (%)	*HoxA11b* (%)	*HoxB1a* (%)	*HoxB4a* (%)	*HoxB1b* (%)	*HoxB5b* (%)	*HoxC4a* (%)	*HoxC6b* (%)	*HoxD4a* (%)	*HoxD10a* (%)
IDC-F1 i: IDC-F1 ii	94.5/98.5	81.8/88.5	93.7/97.6	88.9/96.0	–	92.3/P	94.8/95.7	94.1/96.4	98.0/99.0	92.1/98.4	90.7/96.6	88.0/97.0
: IDC-F1 iii	93.0/98.5	–	96.1/97.9	–	91.5/P	–	–	94.8/96.0	98.5/98.6	–	86.9/94.9	85.8/96.3
: IDC-F1 (1)	96.4/98.0	–	90.1/95.9	–	–	–	–	–	97.5/99.0	–	98.5/99.4	90.3/97.4
: IDC-F1 (2)	–	–	–	–	–	–	–	–	–	–	89.1/95.5	–
: IDC-F1 (BSB)	92.3/98.5	–	–	–	–	–	86.2/91.1	89.4/97.2	–	83.4/97.3	87.8/94.9	81.3/96.7
: IDMC-F1 i	100.0/100.0	99.8/100.0	99.5/98.9	99.8/99.8	99.6/P	99.7/P	100.0/100.0	100.0/100.0	100.0/100.0	100.0/100.0	99.8/99.4	99.9/100.0
: IDMC-F1 ii	94.6/98.5	–	93.8/97.3	–	88.1/P	–	–	94.3/96.8	97.9/98.6	–	90.8/97.2	88.3/97.4
: IDMC-F2 i	100.0/100.0	100.0/100.0	99.6/98.9	99.7/99.7	99.5/P	99.6/P	100.0/100.0	100.0/100.0	99.9/100.0	99.8/100.0	99.8/99.4	99.6/99.6
: IDMC-F2 ii	94.5/98.5	–	93.7/97.3	–	–	–	–	–	97.7/98.6	–	90.9/97.2	88.4/97.4
: IDC-F2-C i	99.7/99.0	99.8/100.0	99.6/98.6	99.9/100.0	99.4/P	99.7/P	100.0/100.0	99.8/100.0	100.0/100.0	99.8/100.0	99.7/98.8	100.0/100.0
: IDC-F2-C ii	94.5/98.5	80.1/86.9	93.7/97.6	–	–	–	–	94.3/96.8	98.0/99.0	–	90.9/97.2	88.5/97.4
: IDC-F2-M i	99.9/100.0	99.8/100.0	99.7/99.3	100.0/100.0	99.5/P	99.7/P	100.0/100.0	99.8/100.0	99.9/100.0	99.8/100.0	99.7/99.4	99.9/100.0
: IDC-F2-M ii	94.2/98.5	–	93.7/97.6	–	–	–	–	94.3/96.4	97.9/99.0	–	90.8/96.6	88.2/97.0
: COC i	99.8/99.0	100.0/100.0	99.6/99.3	99.8/99.7	99.5/P	99.8/P	99.5/100.0	99.9/100.0	99.9/100.0	99.1/98.9	99.6/98.8	99.2/98.5
: COC ii	94.5/98.0	80.2/88.5	93.8/97.6	–	88.2/P	–	–	94.1/96.4	98.0/99.0	–	90.8/97.2	88.3/97.4
: BSB	92.3/98.5	77.3/85.4	94.9/96.9	74.2/94.5	86.7/P	85.9/P	86.2/91.1	89.9/97.6	93.4/91.6	83.3/97.3	87.8/94.9	81.4/97.0
IDC-F1 ii: IDC-F1 iii	96.2/99.5	–	92.6/97.6	–	–	–	–	93.2/95.7	98.4/99.5	–	92.7/96.0	87.9/95.6
: IDC-F1 (1)	93.5/97.5	–	92.3/96.3	–	–	–	–	–	98.7/100.0	–	90.8/97.2	84.0/96.7
: IDC-F1 (2)	–	–	–	–	–	–	–	–	–	–	86.0/94.4	–
: IDC-F1 (BSB)	92.2/98.0	–	–	–	–	–	86.4/89.7	90.0/96.0	–	81.3/96.3	86.2/93.8	81.0/96.3
: IDMC-F1 i	94.5/98.5	81.9/88.5	93.7/97.9	88.8/95.9	–	92.3/P	94.8/95.7	94.1/96.4	98.0/99.0	92.1/98.4	90.8/97.2	88.1/97.0
: IDMC-F1 ii	99.7/100.0	–	99.7/99.6	–	–	–	–	99.1/99.6	99.9/99.5	–	99.4/99.4	99.2/98.9
: IDMC-F2 i	94.5/98.5	81.8/88.5	93.7/97.9	88.9/96.0	–	92.3/P	94.8/95.7	94.1/96.4	97.9/99.0	92.0/98.4	90.8/97.2	87.8/97.4
: IDMC-F2 ii	99.8/100.0	–	99.5/99.6	–	–	–	–	–	99.6/99.5	–	99.5/99.4	99.4/99.6
: IDC-F2-C i	94.4/97.5	81.9/88.5	93.7/97.6	88.9/96.0	–	92.3/P	94.8/95.7	93.9/96.4	98.0/99.0	92.0/98.4	90.7/96.6	88.0/97.0
: IDC-F2-C ii	99.8/100.0	92.6/95.8	99.5/100.0	–	–	–	–	99.3/99.6	100.0/100.0	–	99.5/99.4	99.4/99.6
: IDC-F2-M i	94.6/98.5	81.9/88.5	93.9/98.3	88.9/96.0	–	92.3/P	94.8/95.7	94.1/96.4	98.1/99.0	92.0/98.4	90.7/97.2	88.0/97.0
: IDC-F2-M ii	99.5/100.0	–	99.5/100.0	–	–	–	–	99.1/99.2	99.8/100.0	–	99.4/98.8	99.2/99.2
: COC i	94.5/98.5	81.8/88.5	93.9/98.3	88.9/95.9	–	92.2/P	94.6/95.7	94.0/96.4	98.1/99.0	91.5/97.3	90.8/96.6	87.6/96.3
: COC ii	99.6/99.5	92.1/95.3	99.7/100.0	–	–	–	–	99.1/99.2	100.0/100.0	–	99.3/99.4	99.5/99.6
: BSB	92.2/98.0	81.0/90.1	94.9/97.3	71.6/91.6	–	85.4/P	86.4/89.7	90.4/96.4	93.3/92.5	81.4/96.3	86.2/93.8	81.1/96.7
IDC-F1 iii: IDC-F1 (1)	92.0/97.0	–	89.8/96.3	–	–	–	–	–	98.3/99.5	–	86.9/95.5	84.6/98.1
: IDC-F1 (2)	–	–	–	–	–	–	–	–	–	–	86.0/96.0	–
: IDC-F1 (BSB)	90.8/97.5	–	–	–	–	–	–	88.7/95.7	–	–	84.1/92.7	78.4/95.2
: IDMC-F1 i	93.0/98.5	–	96.1/98.3	–	91.2/P	–	–	94.8/96.0	98.5/98.6	–	87.0/95.5	85.7/96.3
: IDMC-F1 ii	96.2/99.5	–	92.6/97.3	–	84.7/92.6	–	–	93.4/96.0	98.3/99.0	–	92.8/96.6	88.2/95.9
: IDMC-F2 i	93.0/98.5	–	96.2/98.3	–	91.2/P	–	–	94.8/96.0	98.4/98.6	–	87.0/95.5	85.7/96.7
: IDMC-F2 ii	96.2/99.5	–	92.5/97.3	–	–	–	–	–	98.2/99.0	–	92.9/96.6	88.2/95.9
: IDC-F2-C i	92.7/97.5	–	96.2/97.9	–	91.1/P	–	–	94.8/96.0	98.5/98.6	–	86.9/94.9	85.8/96.3
: IDC-F2-C ii	96.2/99.5	–	92.5/97.6	–	–	–	–	93.5/96.0	98.4/99.5	–	92.9/96.6	88.2/95.9
: IDC-F2-M i	92.9/98.5	–	96.3/98.6	–	91.2/P	–	–	94.6/96.0	98.6/98.6	–	86.9/95.5	85.8/96.3
: IDC-F2-M ii	95.9/99.5	–	92.5/97.6	–	–	–	–	93.4/95.7	98.3/99.5	–	92.8/96.0	88.1/95.6
: COC i	93.0/98.0	–	96.2/98.6	–	91.2/P	–	–	94.7/96.0	98.6/98.6	–	87.0/94.9	85.4/95.6
: COC ii	96.2/99.0	–	92.6/97.6	–	84.9/92.6	–	–	93.4/95.7	98.4/99.5	–	92.7/96.6	88.1/95.9
: BSB	90.8/97.5	–	93.5/97.3	–	83.4/94.2	–	–	88.8/96.0	93.3/92.1	–	84.1/92.7	78.5/95.6
IDC-F1 (1): IDC-F1 (2)	–	–	–	–	–	–	–	–	–	–	88.8/96.0	–
: IDC-F1 (BSB)	91.3/97.5	–	–	–	–	–	–	–	–	–	87.8/95.5	78.1/96.3
: IDMC-F1 i	96.4/98.0	–	90.1/96.3	–	–	–	–	–	97.5/99.0	–	98.6/100.0	90.3/97.4
: IDMC-F1 ii	93.5/97.5	–	92.4/95.9	–	–	–	–	–	98.6/99.5	–	90.9/97.7	84.2/97.0
: IDMC-F2 i	96.4/98.0	–	90.2/96.3	–	–	–	–	–	97.4/99.0	–	98.6/100.0	90.2/97.8
: IDMC-F2 ii	93.5/97.5	–	92.3/95.9	–	–	–	–	–	98.3/99.5	–	91.0/97.7	84.2/97.0
: IDC-F2-C i	96.1/97.0	–	90.2/95.9	–	–	–	–	–	97.5/99.0	–	98.5/99.4	90.3/97.4
: IDC-F2-C ii	93.5/97.5	–	92.3/96.3	–	–	–	–	–	98.7/100.0	–	91.0/97.7	84.3/97.0
: IDC-F2-M i	96.3/98.0	–	90.3/96.6	–	–	–	–	–	97.6/99.0	–	98.5/100.0	90.3/97.4
: IDC-F2-M ii	93.2/97.5	–	92.3/96.3	–	–	–	–	–	98.5/100.0	–	90.9/97.2	84.1/96.7
: COC i	96.4/98.0	–	90.2/96.6	–	–	–	–	–	97.6/99.0	–	98.6/99.4	90.1/96.7
: COC ii	93.5/97.0	–	92.4/96.3	–	–	–	–	–	98.7/100.0	–	90.9/97.7	84.2/97.0
: BSB	91.3/97.5	–	91.0/96.3	–	–	–	–	–	93.4/92.5	–	87.8/95.5	78.2/96.7
IDC-F1 (2): IDC-F1 (BSB)	–	–	–	–	–	–	–	–	–	–	83.5/94.4	–
: IDMC-F1 i	–	–	–	–	–	–	–	–	–	–	89.2/96.0	–
: IDMC-F1 ii	–	–	–	–	–	–	–	–	–	–	86.2/94.9	–
: IDMC-F2 i	–	–	–	–	–	–	–	–	–	–	89.2/96.0	–
: IDMC-F2 ii	–	–	–	–	–	–	–	–	–	–	86.3/94.9	–
: IDC-F2-C i	–	–	–	–	–	–	–	–	–	–	89.1/95.5	–
: IDC-F2-C ii	–	–	–	–	–	–	–	–	–	–	86.3/94.9	–
: IDC-F2-M i	–	–	–	–	–	–	–	–	–	–	89.1/96.0	–
: IDC-F2-M ii	–	–	–	–	–	–	–	–	–	–	86.2/94.4	–
: COC i	–	–	–	–	–	–	–	–	–	–	89.2/95.5	–
: COC ii	–	–	–	–	–	–	–	–	–	–	86.1/94.9	–
: BSB	–	–	–	–	–	–	–	–	–	–	83.5/94.4	–
IDC-F1 (BSB): IDMC-F1 i	92.3/98.5	–	–	–	–	–	86.2/91.1	89.4/97.2	–	83.4/97.3	87.9/95.5	81.4/96.7
: IDMC-F1 ii	92.3/98.0	–	–	–	–	–	–	90.0/96.4	–	–	86.5/94.4	81.3/96.7
: IDMC-F2 i	92.3/98.5	–	–	–	–	–	86.2/91.1	89.4/97.2	–	83.4/97.3	87.9/95.5	81.2/97.0
: IDMC-F2 ii	92.2/98.0	–	–	–	–	–	–	–	–	–	86.6/94.4	81.3/96.7
: IDC-F2-C i	92.0/97.5	–	–	–	–	–	86.2/91.1	89.4/97.2	–	83.5/97.3	87.8/94.9	81.3/96.7
: IDC-F2-C ii	92.2/98.0	–	–	–	–	–	–	90.2/96.4	–	–	86.6/94.4	81.3/96.7
: IDC-F2-M i	92.2/98.5	–	–	–	–	–	86.2/91.1	89.3/97.2	–	83.3/97.3	87.8/95.5	81.3/96.7
: IDC-F2-M ii	91.9/98.0	–	–	–	–	–	–	90.0/96.0	–	–	86.5/93.8	81.2/96.3
: COC i	92.3/98.5	–	–	–	–	–	86.0/91.1	89.4/97.2	–	82.7/96.3	87.9/94.9	80.9/95.9
: COC ii	92.2/97.5	–	–	–	–	–	–	90.0/96.0	–	–	86.5/94.4	81.3/96.7
: BSB	100.0/100.0	–	–	–	–	–	100.0/100.0	98.4/99.6	–	99.5/100.0	100.0/100.0	99.8/99.6

Notes: Values before slashes (/) denote nucleotide identity, and values after slashes denote amino acid identity; P represents one or two amino acid sequences as pseudogene sequences for which the identity cannot be compared

**Table 4:** Percentage nucleotide identity (on the left) and percentage amino acid identity (on the right) between duplicated *Hox* coding regions in COC, BSB, IDC-F_1_, IDMC-F_1_, IDMC-F_2_, IDC-F_2_-C, and IDC-F_2_-M (following the format above)

	HoxA4a (%)	HoxA9a (%)	HoxA2b (%)	HoxA11b (%)	HoxB1a (%)	HoxB4a (%)	HoxB1b (%)	HoxB5b (%)	HoxC4a (%)	HoxC6b (%)	HoxD4a (%)	HoxD10a (%)
IDMC-F1 i: IDMC-F1 ii	94.6/98.5	–	93.8/97.6	–	87.7/P	–	–	94.3/96.8	97.9/98.6	–	90.9/97.7	88.4/97.4
: IDMC-F2 i	100.0/100.0	99.8/100.0	99.6/99.3	99.7/99.8	99.6/P	99.6/P	100.0/100.0	100.0/100.0	99.9/100.0	99.8/100.0	100.0/100.0	99.5/99.6
: IDMC-F2 ii	94.5/98.5	–	93.7/97.6	–	–	–	–	–	97.7/98.6	–	91.0/97.7	88.5/97.4
: IDC-F2-C i	99.7/99.0	100.0/100.0	99.6/98.9	99.7/99.8	99.5/P	99.8/P	100.0/100.0	99.8/100.0	100.0/100.0	99.8/100.0	99.8/99.4	99.9/100.0
: IDC-F2-C ii	94.5/98.5	80.2/86.9	93.8/97.9	–	–	–	–	94.3/96.8	98.0/99.0	–	91.0/97.7	88.6/97.4
: IDC-F2-M i	99.9/100.0	100.0/100.0	99.7/99.6	99.8/99.8	99.6/P	99.8/P	100.0/100.0	99.8/100.0	99.9/100.0	99.8/100.0	99.8/100.0	99.8/100.0
: IDC-F2-M ii	94.2/98.5	–	93.8/97.9	–	–	–	–	94.3/96.4	97.9/99.0	–	90.9/97.2	88.3/97.0
: COC i	99.8/99.0	99.8/100.0	99.7/99.6	99.7/99.5	99.5/P	99.6/P	99.5/100.0	99.9/100.0	99.9/100.0	99.1/98.9	99.7/99.4	99.2/98.5
: COC ii	94.5/98.0	80.1/88.5	93.8/97.9	–	87.8/P	–	–	94.1/96.4	98.0/99.0	–	90.9/97.7	88.3/97.4
: BSB	92.3/98.5	77.2/85.4	95.1/97.3	74.2/94.4	86.3/P	85.8/P	86.2/91.1	89.9/97.6	93.4/91.6	83.3/97.3	87.9/95.5	81.5/97.0
IDMC-F1 ii: IDMC-F2 i	94.6/98.5	–	93.9/97.6	–	87.8/P	–	–	94.3/96.8	97.8/98.6	–	90.9/97.7	88.1/97.8
: IDMC-F2 ii	99.9/100.0	–	99.7/99.3	–	–	–	–	–	99.5/99.0	–	99.8/100.0	99.6/99.2
: IDC-F2-C i	94.5/97.5	–	93.9/97.3	–	87.6/P	–	–	94.3/96.8	97.9/98.6	–	90.8/97.2	88.3/97.4
: IDC-F2-C ii	99.9/100.0	–	99.7/99.6	–	–	–	–	99.8/100.0	99.9/99.5	–	99.8/100.0	99.6/99.2
: IDC-F2-M i	94.5/98.5	–	94.0/97.9	–	87.8/P	–	–	94.3/96.8	98.0/98.6	–	90.8/97.7	88.3/97.4
: IDC-F2-M ii	99.6/100.0	–	99.7/99.6	–	–	–	–	99.8/99.6	99.7/99.5	–	99.7/99.4	99.2/98.9
: COC i	94.6/98.5	–	93.9/97.9	–	87.8/P	–	–	94.2/96.8	98.0/98.6	–	90.9/97.2	88.0/96.7
: COC ii	99.7/99.5	–	99.8/99.6	–	99.2/99.1	–	–	99.8/99.6	99.9/99.5	–	99.6/100.0	99.4/99.2
: BSB	92.3/98.0	–	95.0/96.9	–	85.0/95.9	–	–	90.5/96.8	93.3/92.1	–	86.5/94.4	81.4/97.0
IDMC-F2 i: IDMC-F2 ii	94.5/98.5	–	93.7/97.6	–	–	–	–	–	97.8/98.6	–	91.0/97.7	88.2/97.8
: IDC-F2-C i	99.7/99.0	99.8/100.0	99.7/98.9	99.7/99.7	99.8/P	99.6/P	100.0/100.0	99.8/100.0	99.9/100.0	99.7/100.0	99.8/99.4	99.6/99.6
: IDC-F2-C ii	94.5/98.5	80.1/86.9	93.7/97.9	–	–	–	–	94.3/96.8	97.9/99.0	–	91.0/97.7	88.3/97.8
: IDC-F2-M i	99.9/100.0	99.8/100.0	99.8/99.6	99.7/99.7	100.0/P	99.6/P	100.0/100.0	99.8/100.0	99.8/100.0	99.7/100.0	99.8/100.0	99.6/99.6
: IDC-F2-M ii	94.2/98.5	–	93.7/97.9	–	–	–	–	94.3/96.4	97.8/99.0	–	90.9/97.2	87.9/97.4
: COC i	99.8/99.0	100.0/100.0	99.7/99.6	99.7/99.5	99.8/P	99.6/P	99.5/100.0	99.9/100.0	99.8/100.0	99.0/98.9	99.7/99.4	99.1/98.9
: COC ii	94.5/98.0	80.2/88.5	93.9/97.9	–	87.8/P	–	–	94.1/96.4	97.9/99.0	–	90.9/97.7	88.0/97.8
: BSB	92.3/98.5	77.3/85.4	95.0/97.3	74.0/94.2	86.4/P	85.9/P	86.2/91.1	89.9/97.6	93.5/91.6	83.3/97.3	87.9/95.5	81.3/97.4
IDMC-F2 ii: IDC-F2-C i	94.4/97.5	–	93.7/97.3	–	–	–	–	–	97.7/98.6	–	90.9/97.2	88.4/97.4
: IDC-F2-C ii	100.0/100.0	–	99.7/99.6	–	–	–	–	–	99.6/99.5	–	100.0/100.0	99.8/100.0
: IDC-F2-M i	94.6/98.5	–	93.9/97.9	–	–	–	–	–	97.8/98.6	–	90.9/97.7	88.3/97.4
: IDC-F2-M ii	99.7/100.0	–	99.7/99.6	–	–	–	–	–	99.8/99.5	–	99.8/99.4	99.4/99.6
: COC i	94.5/98.5	–	93.7/97.9	–	–	–	–	–	97.8/98.6	–	91.0/97.2	88.0/96.7
: COC ii	99.6/99.5	–	99.7/99.6	–	–	–	–	–	99.6/99.5	–	99.7/100.0	99.6/100.0
: BSB	92.2/98.0	–	94.9/96.9	–	–	–	–	–	93.2/92.1	–	86.6/94.4	81.4/97.0
IDC-F2-C i: IDC-F2-C ii	94.4/97.5	80.2/86.9	93.7/97.6	–	–	–	–	94.3/96.8	98.0/99.0	–	90.9/97.2	88.5/97.4
: IDC-F2-M i	99.6/99.0	100.0/100.0	99.8/99.3	99.9/100.0	99.8/P	99.8/P	100.0/100.0	99.8/100.0	99.9/100.0	99.7/100.0	99.7/99.4	99.9/100.0
: IDC-F2-M ii	94.1/97.5	–	93.7/97.6	–	–	–	–	94.3/96.4	97.9/99.0	–	90.8/96.6	88.2/97.0
: COC i	99.5/98.0	99.8/100.0	99.7/99.3	99.7/99.7	99.7/P	99.6/P	99.5/100.0	99.7/100.0	99.9/100.0	99.1/98.9	99.6/98.8	99.2/98.5
: COC ii	94.4/97.0	80.1/88.5	93.9/97.6	–	87.7/P	–	–	94.1/96.4	98.0/99.0	–	90.8/97.2	88.3/97.4
: BSB	92.0/97.5	77.2/85.4	95.0/96.9	74.2/94.5	86.3/P	85.8/P	86.2/91.1	89.7/97.6	93.4/91.6	83.2/97.3	87.8/94.9	81.4/97.0
IDC-F2-C ii: IDC-F2-M i	94.6/98.5	80.2/86.9	93.9/98.3	–	–	–	–	94.3/96.8	98.1/99.0	–	90.9/97.7	88.5/97.4
: IDC-F2-M ii	99.7/100.0	–	100.0/100.0	–	–	–	–	99.8/99.6	99.8/100.0	–	99.8/99.4	99.4/99.6
: COC i	94.5/98.5	80.1/86.9	93.9/98.3	–	–	–	–	94.2/96.8	98.1/99.0	–	91.0/97.2	88.2/96.7
: COC ii	99.6/99.5	99.2/98.4	99.7/100.0	–	–	–	–	99.8/99.6	100.0/100.0	–	99.7/100.0	99.6/100.0
: BSB	92.2/98.0	80.3/88.5	95.0/97.3	–	–	–	–	90.5/96.8	93.3/92.5	–	86.6/94.4	81.4/97.0
IDC-F2-M i: IDC-F2-M ii	94.3/98.5	–	93.9/98.3	–	–	–	–	94.3/96.4	98.0/99.0	–	90.8/97.2	88.1/97.0
: COC i	99.7/99.0	99.8/100.0	99.8/100.0	99.8/99.7	99.8/P	99.6/P	99.5/100.0	99.7/100.0	100.0/100.0	99.0/98.9	99.6/99.4	99.2/98.5
: COC ii	94.4/98.0	80.1/88.5	94.0/98.3	–	87.8/P	–	–	94.1/96.4	98.1/99.0	–	90.8/97.7	88.2/97.4
: BSB	92.2/98.5	77.2/85.4	95.1/97.6	74.2/94.5	86.4/P	85.8/P	86.2/91.1	89.7/97.6	93.5/91.6	83.2/97.3	87.8/95.5	81.4/97.0
IDC-F2-M ii: COC i	94.2/98.5	–	93.9/98.3	–	–	–	–	94.2/96.4	98.0/99.0	–	90.9/96.6	87.8/96.3
: COC ii	99.4/99.5	–	99.7/100.0	–	–	–	–	99.6/99.2	99.8/100.0	–	99.6/99.4	99.4/99.6
: BSB	91.9/98.0	–	95.0/97.3	–	–	–	–	90.5/96.4	93.3/92.5	–	86.5/93.8	81.3/96.7
COC i: COC ii	94.5/98.0	80.2/88.5	93.9/98.3	–	87.8/P	–	–	94.0/96.4	98.1/99.0	–	90.9/97.2	87.9/96.7
: BSB	92.3/98.5	77.3/85.4	95.1/97.6	74.2/94.5	86.4/P	86.0/P	86.0/91.1	89.8/97.6	93.5/91.6	82.9/96.3	87.9/94.9	81.0/96.3
COC ii: BSB	92.2/97.5	80.3/89.0	95.0/97.3	–	85.2/95.9	–	–	90.3/96.4	93.3/92.5	–	86.5/94.4	81.4/97.0

Notes: Values before slashes (/) denote nucleotide identity, and values after slashes denote amino acid identity; P represents one or two amino acid sequences as pseudogene sequences for which the identity cannot be compared

## Discussion

Hybridization offers a means by which diversity may be increased because, unlike mutation, it provides genetic variation at hundreds or thousands of genes in a single generation [4]. Our results provide a good model for genetic variation by showing obvious genotypic differences in the IDC-F_1_ fish derived from the distant hybridization of COC (♀) × BSB (♂). The *Hox* gene clusters in IDC-F_1_ were approximately twice as large as those in COC, except for the recombinant clusters. The topology of the phylogenetic tree of 12 *Hox* genes (Fig. 3) further suggested that some of the *Hox* genes orthologous to zebrafish genes were present as two copies in COC (except for *HoxA11b*, *HoxB1b*, *HoxB4a*, and *HoxC6b*), one copy in BSB, and two to six copies (not counting recombinant clusters) in IDC-F_1_. The proliferation of such a rich diversity in gene copy number further reveals that distant hybridization as a catalyst accelerates the formation of species [8]. One of the highlights of this study is the development of IDMC-F_1_ derived from the distant hybridization of COC (♀) × BSB (♂), which has a significant difference in phenotype compared to its parents; even in the self-crossed offspring of IDC-F_1_, two distinct phenotypes were differentiated: IDC-F_2_-C was consistent with that of IDC-F_1_, and IDC-F_2_-M was very similar to that of IDMC-F_1_ (Fig. 1). Determining the mechanisms that lead to these new phenotypes to appear will help us to understand the impact of hybridization on the speciation processes. At present, three possible mechanisms are considered. Firstly, alleles of additive effect may not all be fixed in the same direction between diverging populations, under this mechanism, some hybrid genotypes then fall outside the parental distribution (+ + + − × − − − + can generate + + + + or − − − −) [30]. Secondly, these new phenotypes derived from hybridization may result from interactions (dominance or epistasis) between alleles fixed independently in different populations. Thirdly, research in recent years has begun to reveal a wider variety of genetic mechanisms underlying new hybrid phenotypes, e.g., genome restructuring, duplication/deletion [31], alterations in the timing and levels of gene expression, transposon activation and epigenetic effects [32–35]. We speculated that the third mechanism was the possible reason for the differentiation of the mirror carp-type offspring (IDMC-F_1_ and IDC-F_2_-M). Under this mechanism, the genomes of hybrid progeny contain a rich variety of genetic variants, which are rapidly changing in the early generation of hybridization, and most of the variant types cannot be stably inherited to the next generation. In fact, most of the *Hox* gene copies in IDC-F_1_ were not stably inherited in the second generation (IDC-F_2_-C and IDC-F_2_-M). These result validated the possible mechanism of the differentiation of the mirror carp-type offspring. In contrast, as with the first generation of distant hybridization, we did not find obvious mutations in *Hox* genes in the first generation of IDMC; almost all of the *Hox* gene clusters were derived from the female parent, COC, except for the recombinant clusters. Almost all of the *Hox* gene clusters in IDMC-F_1_ were stably inherited in IDMC-F_2_, but at the same time, IDMC-F_2_ contained more obvious recombination events. The gene types that were stably inherited from a single parent in the offspring have experienced long-term evolutionary testing and became essential for the evolution of species.

The functions of *Hox* genes have become increasingly clear in recent years, but questions about the evolution of *Hox* genes remain unresolved. Gene duplication and mutation are the basis for understanding *Hox* gene evolution, and mutations in coding sequences may produce new functional proteins. *Hox* gene clusters in fish are more variable in gene content than expected, and each cluster has its own characteristics in terms of absolute length and content of conserved non-coding sequences [36]. This study fully confirms this argument; for example, among these *Hox* genes, two to six copies (not counting recombinant clusters) were found in IDC-F_1_. Furthermore, *Hox* cluster degeneration may be ongoing, at least in fish, because *HoxB4a* is active in zebrafish but its orthologues are pseudogenes in COC, BSB, IDC-F_1_, IDMC-F_1_, IDC-F_2_-C, IDC-F_2_-M, and IDMC-F_2_. Similarly, *HoxB1ai* is active in zebrafish, but its orthologues are pseudogenes in COC, IDC-F_1_, IDMC-F_1_, IDC-F_2_-C, IDC-F_2_-M, and IDMC-F_2_. These results revealed that the *Hox* gene clusters are undergoing continuous degeneration in the cyprinid fishes, with some genes gradually becoming pseudogenes, and some genes completely pseudogenised.

One of the most important finding of this study is the discovery of *Hox* gene recombinant clusters, which may be the first in *Hox* genes of cyprinid fishes or even vertebrates. In the two types of improved carp lineages derived from COC (♀) × BSB (♂), these recombinant clusters come from the recombination of different types of gene copies, most of which cannot be stably inherited to the next generation. Moreover, for *HoxA11b*, we found the recombinant cluster type (*HoxA11bi* + *HoxA11b-BSB* + *HoxA11bi*) only in IDC-F_2_-M and IDMC-F_2_, indicating that it might be necessary for development of the morphological features of mirror carp-like species. In this study, we studied the genetic variation in 12 *Hox* genes in the two types of improved carp lineages derived from COC (♀) × BSB (♂). We first revealed the interesting results of the abundant gene clusters derived from IDC-F_1_ and found a wide variety of recombinant clusters in the two types of improved carp lineages. In summary, our results provided important evidence that distant hybridization produced rapid genomic DNA changes that may or may not stably inherited, providing novel insight into the function of hybridization in the establishment of the improved lineages used as new fish resources for aquaculture. The genetic evolution of the *Hox* gene family provides clues for revealing the gene regulatory mechanisms underlying biological evolution and cell differentiation.

## Conclusions

Based on the establishment of the two types of improved carp lineages derived from common carp (♀) × blunt snout bream (♂), our results provided important evidence that distant hybridization produced rapid genomic DNA changes that may or may not stably inherited, providing novel insight into the function of hybridization in the establishment of the improved lineages used as new fish resources for aquaculture.

## Methods

### Ethics statement

The guidelines established by the Administration of Affairs Concerning Animal Experimentation state that approval from the Science and Technology Bureau of China and the Department of Wildlife Administration is not necessary when the fish in question are neither rare nor near extinction (first- or second-class state protection level). Therefore, approval was not required for the experiments conducted in this study.

### Animals and crossing procedure

All of the natural materials, such as common carp (*Cyprinus carpio*, 2n = 100, abbreviated as COC) and blunt snout bream (*Megalobrama amblycephala*, 2n = 48, abbreviated as BSB) were obtained from the Center for Polyploidy Fish Genetics Breeding of Hunan Province located at Hunan Normal University, Changsha, Hunan, China. The protocols for crossing and culturing were described previously [23]. The two types of improved carp offspring from COC (♀) × BSB (♂) were the improved diploid common carp (2n = 100, IDC-F_1_) and the improved diploid scattered mirror carp (2n = 100, IDMC-F_1_); the phenotype of the latter has changed significantly from that of the female parent, COC. The self-crossed offspring of IDC-F_1_ showed two phenotypes: one was consistent with that of their parents (abbreviated IDC-F_2_-C), and the other was very similar to that of IDMC-F_1_ (abbreviated IDC-F_2_-M). In contrast, the self-crossed offspring of IDMC-F_1_ showed only one phenotype, that is, a scattered mirror carp-like appearance (abbreviated IDMC-F_2_). The IDC-F_1_, IDMC-F_1_, IDC-F_2_-C, IDC-F_2_-M, and IDMC-F_2_ fish were cultured in ponds at the Center for Polyploidy Fish Genetics Breeding of Hunan Province located at Hunan Normal University, Changsha, Hunan, China, and fed artificial feed. All fishes were deeply anaesthetized with 100 mg/L MS-222 (Sigma-Aldrich, St. Louis, MO, USA) prior to dissection.

### DNA extraction, PCR amplification, cloning and sequencing of *Hox* genes

Total genomic DNA from the peripheral blood cells of COC, BSB, IDC-F_1_, IDMC-F_1_, IDC-F_2_-C, IDC-F_2_-M, and IDMC-F_2_ extracted by routine approaches [37] were used separately as templates. Several combinations of degenerate PCR primers (Additional file 1: Table S1) [38, 39] were used to amplify up to 12 *Hox* gene sequences (*HoxA4a*, *HoxA9a*, *HoxA2b*, *HoxA11b*, *HoxB1a*, *HoxB4a*, *HoxB1b*, *HoxB5b*, *HoxC4a*, *HoxC6b*, *HoxD4a*, and *HoxD10a*) in COC, BSB, IDC-F_1_, IDMC-F_1_, IDC-F_2_-C, IDC-F_2_-M, and IDMC-F_2_. The PCRs were performed in a volume of 50 μL using Taq DNA polymerase (TaKaRa, Dalian, China). The thermal cycling program uses thermal gradient PCR and used these conditions for the first time. The thermal cycling program generally consisted of an initial denaturation step at 94 °C for 5 min, followed by 35 cycles of 94 °C for 35 s, 50–60 °C for 60 s, and 72 °C for 60–150 s and a final extension step at 72 °C for 10 min. The PCR products were cloned into the pMD18-T vector (TaKaRa, Dalian, China). The plasmids were transformed into *E. coli* DH5a, purified and sequenced with vector-specific primers using the primer walking method on an ABI 3730XL automatic sequencer (ABI PRISM 3730, Applied Biosystems, CA, USA). The sequences were BLAST searched against the non-redundant protein database maintained at the National Center for Biotechnology Information (www.ncbi.nlm.nih.gov) to determine their identity.

### Sequence comparison and analysis

All of the sequence information and GenBank accession numbers in this study is detailed in Additional file 1: Table S2. The sequence homology and variation among the fragments amplified from COC, BSB, IDC-F_1_, IDMC-F_1_, IDC-F_2_-C, IDC-F_2_-M, and IDMC-F_2_ were analysed using BioEdit [40] and the DNAStar 5.0 software package (DNAStar Inc.). To increase the probability of detecting duplicated paralogs and circumventing errors from PCR, we sequenced 20–30 clones for each gene from each of COC, BSB, IDC-F_1_, IDMC-F_1_, IDC-F_2_-C, IDC-F_2_-M, and IDMC-F_2_. The obtained sequences were screened for *Hox* gene fragments using the BLAST (http://www.ncbi.nlm.nih.gov), ClustalW (http://www.ebi.ac.uk/) [41] and MEGA 4.0 [42] programs to determine identity. Then, we evaluated the organization of the *Hox* clusters in IDC-F_1_, IDMC-F_1_, IDC-F_2_-C, IDC-F_2_-M, and IDMC-F_2_ in comparison with COC and BSB to characterize the *Hox* genes.

### Phylogenetic analysis - unconstrained Bayesian analysis

The derived amino acid sequences of 12 *Hox* genes (*HoxA4a*, *HoxA9a*, *HoxA2b*, *HoxA11b*, *HoxB1a*, *HoxB4a*, *HoxB1b*, *HoxB5b*, *HoxC4a*, *HoxC6b*, *HoxD4a*, and *HoxD10a*) in COC, BSB, IDC-F_1_, IDMC-F_1_, IDC-F_2_-C, IDC-F_2_-M, IDMC-F_2_ were aligned with the *Hox* genes of zebrafish retrieved from GenBank using Clustal X 1.81 [43]. Regions of zebrafish *Hox* gene sequence that were difficult to align were removed from the alignment. Gaps were also removed from the alignment. An unrooted phylogenetic tree of all amino acid sequences of the 12 *Hox* genes (the pseudogenes found in this study were not excluded from the phylogenetic analysis) was analysed in MrBayes version 3.1.2 [44, 45]. We also tested the *Hox* genes for saturation using DAMBE v6.4.41 [46], and the results revealed that the *Hox* genes were suitable for phylogenetic analysis. The best-fitting substitution models for each gene fragment were determined by Modeltest 3.7 [47], and the HKY + I + G model was chosen for the *Hox* genes by using the Bayesian information criterion. MrBayes was run for 2 million generations with two runs and four chains in parallel and a burn-in of 25%, and the analysis was terminated after the average standard deviation of the split frequencies fell under 0.01. The final trees were visualized in FIGTREE 1.4.4 (http://tree.bio.ed.ac.uk/software/figtree/. 2018.).

## Supplementary information

**Additional file 1: Table S1.** The 12 combinations of degenerate PCR primers designed based on the alignment and identification of consensus sequences of orthologous *Hox* genes from zebrafish (*Danio rerio*), medaka (Oryzias latipes), rainbow trout (Oncorhynchus mykiss), pufferfish (Fugu rubripes), mouse (Mus musculus), and humans (Homo sapiens). **Table S2.** Sequence information and GenBank accession numbers for COC, BSB, IDC-F1, IDMC-F1, IDC-F2-C, IDC-F2-M, and IDMC-F2 clones, and GenBank accession numbers of zebrafish (*Danio rerio*) used in this study; the tick symbol means the suquence used in this analysis.

**Additional file 2: Figure S1.** Variable sequence types (including haplotypes and recombinant clusters) in different *Hox* genes in these species. **Figure S2.** Variable sequence types (including haplotypes and recombinant clusters) in different *Hox* genes in these species. **Figure S3.** Variable sequence types (including haplotypes and recombinant clusters) in *HoxB5b* in these species.

## Data Availability

The sequencing data are deposited in NCBI (http://www.ncbi.nlm/.nih.gov/nuccore) and is available from the authors. All of the sequence information and GenBank accession numbers in this study is detailed in Additional file 1: Table S2. The fish cannot be made publicly available, because they are property of the Hunan Normal University.

## Abbreviations

BSB: Blunt snout bream (*Megalobrama amblycephala*)
COC: Common carp (*Cyprinus carpio*)
IDC-F_1_: The first generation of the improved diploid common carp derived from common carp (♀) × blunt snout bream (♂)
IDC-F_2_-C: The self-crossed offspring of IDC-F_1_ (common carp-type)
IDC-F_2_-M: The self-crossed offspring of IDC-F_1_ (mirror carp-type)
IDMC-F_1_: The first generation of the improved diploid scattered mirror carp derived from common carp (♀) × blunt snout bream (♂)
IDMC-F_2_: The self-crossed offspring of IDMC-F_1_
