# Supplementary material for: Rapid genomic DNA variation in newly hybridized carp lineages derived from Cyprinus carpio (♀) × Megalobrama amblycephala (♂)
Source: BMC Genet. 2019 Nov 28;20:87. doi: 10.1186/s12863-019-0784-2 (PMC6883602; doi:10.1186/s12863-019-0784-2)
Supplement: Supplementary file 2 — Additional file 2: Figure S1. Variable sequence types (including haplotypes and recombinant clusters) in different Hox genes in these species. Figure S2. Variable sequence types (including haplotypes and recombinant clusters) in different Hox genes in these species. Figure S3. Variable sequence types (including haplotypes and recombinant clusters) in HoxB5b in these species. [file 12863_2019_784_MOESM2_ESM.docx]

**Supplementary Information Figures**


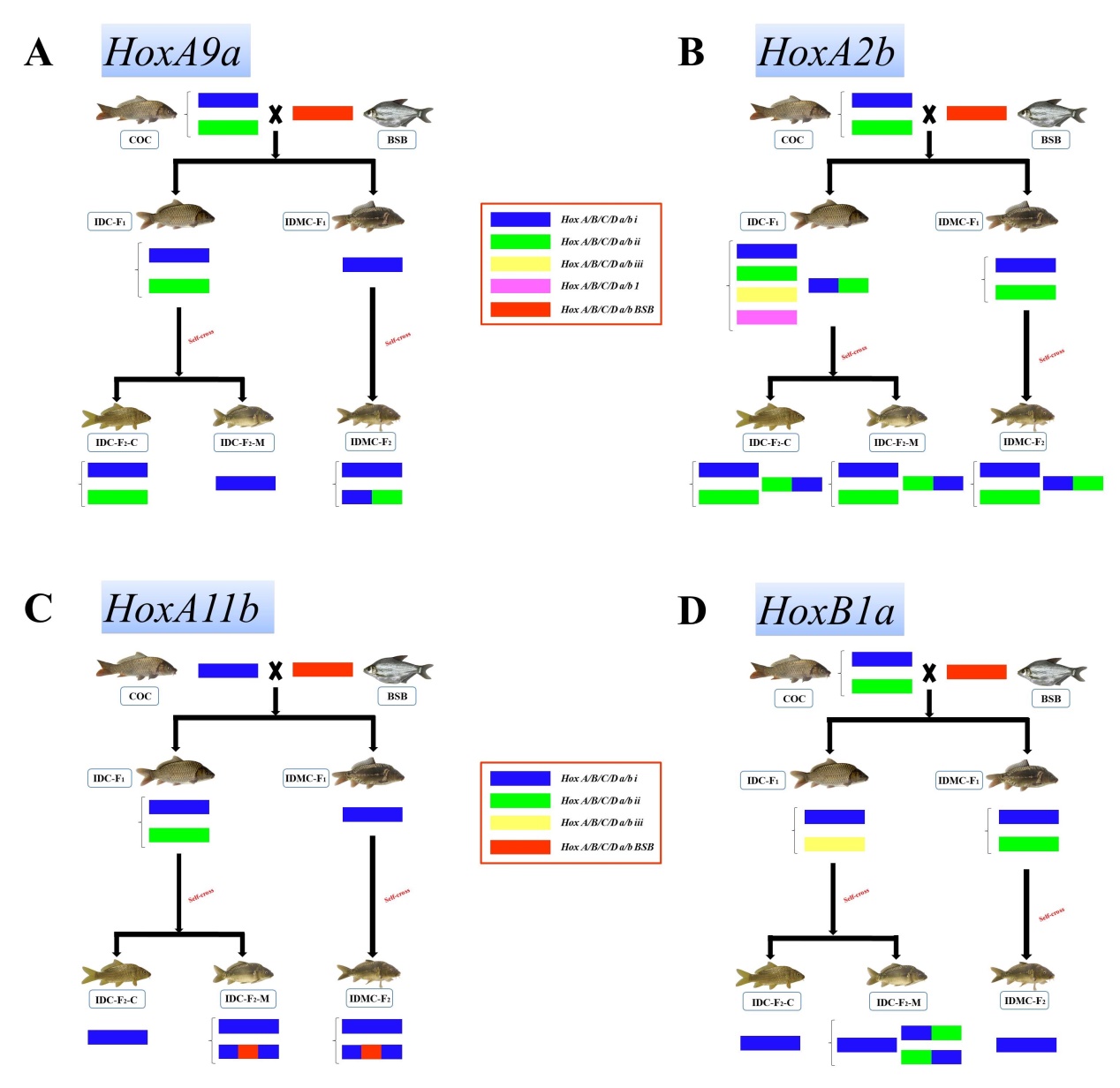


**Figure S1. Variable sequence types (including haplotypes and recombinant clusters) in different *Hox* genes in these species.** a: Variable sequence types (including haplotypes and recombinant clusters) in *HoxA9a* in these species. b: Variable sequence types (including haplotypes and recombinant clusters) in *HoxA2b* in these species. c: Variable sequence types (including haplotypes and recombinant clusters) in *HoxA11b* in these species. d: Variable sequence types (including haplotypes and recombinant clusters) in *HoxB1a* in these species.

**
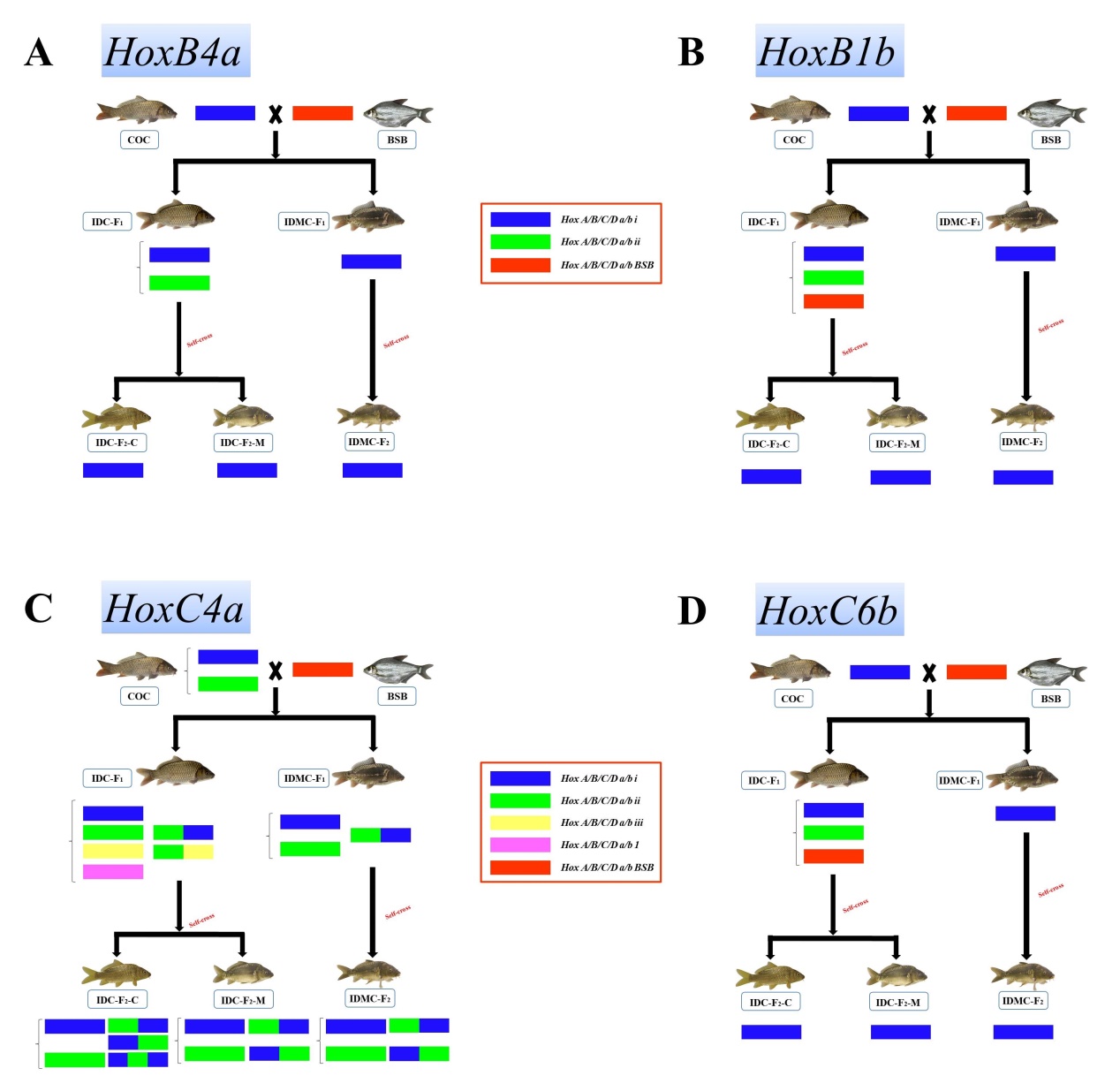
**

**Figure S2. Variable sequence types (including haplotypes and recombinant clusters) in different *Hox* genes in these species.** a: Variable sequence types in *HoxB4a* in these species. b: Variable sequence types in *HoxB1b* in these species. c: Variable sequence types (including haplotypes and recombinant clusters) in *HoxC4a* in these species. d: Variable sequence types in *HoxC6b* in these species.

**
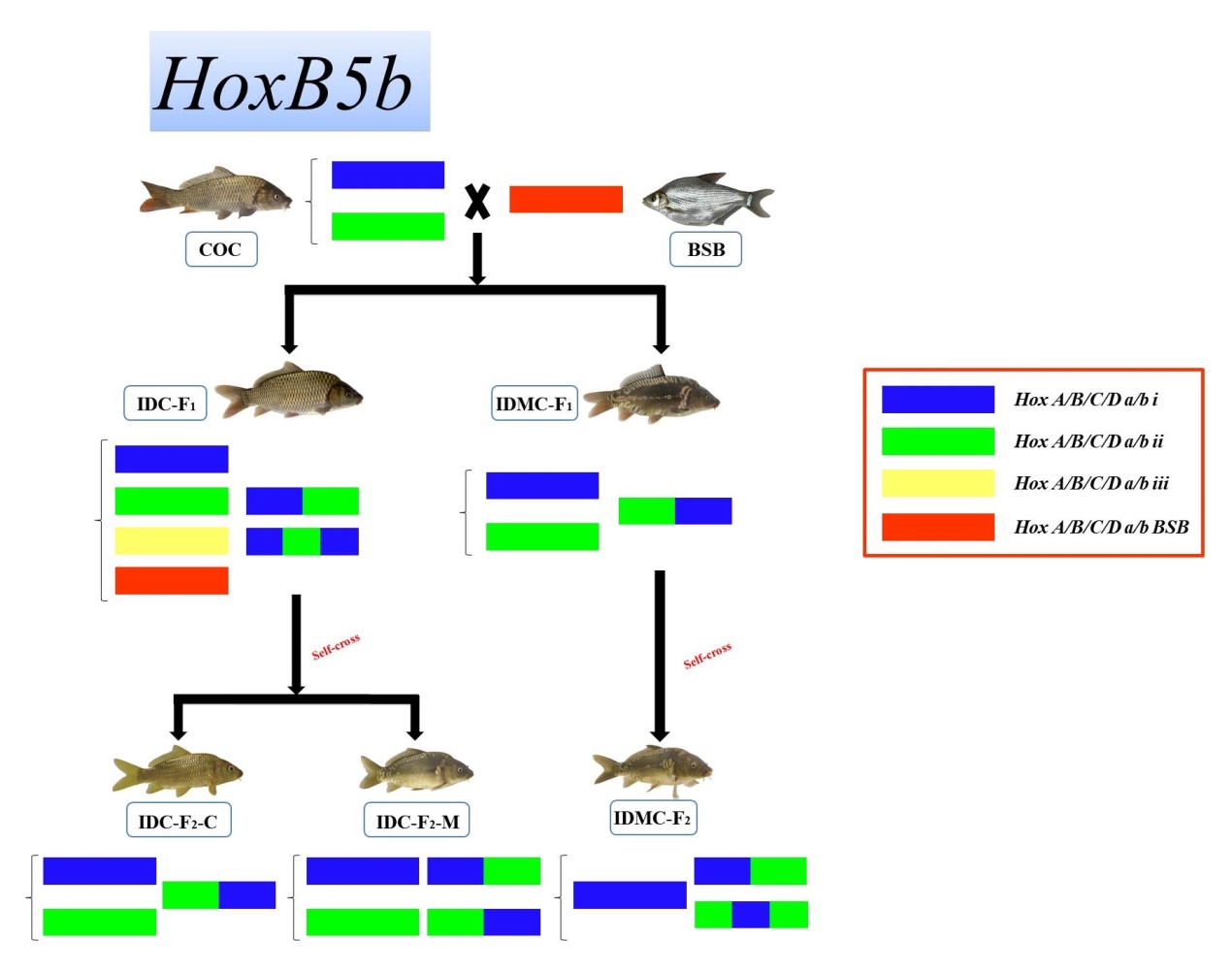
**

**Figure S3. Variable sequence types (including haplotypes and recombinant clusters) in *HoxB5b* in these species.**
